# Development and Validation of a Scoring System for Prediction of Tolerance to Inhaled Treprostinil in Patients with PAH or PH-ILD

**DOI:** 10.3390/jcm14186395

**Published:** 2025-09-10

**Authors:** Alan Lanurias Diaz, Ashwin Kumar, Obada Kholoki, David M. O’Sullivan, Kristen Swanson, Brett Carollo, Joseph Bahgat, Harrison W. Farber, Raj Parikh

**Affiliations:** 1 Department of Internal Medicine, University of Connecticut, Farmington, CT 06030, USA; ashwinkumar1351@gmail.com (A.K.); kholoki@uchc.edu (O.K.); 2 Department of Research Administration, Hartford HealthCare, Hartford, CT 06102, USA; 3 Division of Pulmonary, Critical Care, and Sleep, Hartford Hospital, Hartford, CT 06102, USA; kristen.swanson@hhchealth.org (K.S.); brett.carollo@hhchealth.org (B.C.);; 4 Division of Pulmonary and Critical Care, University of Connecticut, Farmington, CT 06030, USA; jabags@att.net; 5 Division of Pulmonary, Sleep and Critical Care Medicine, Tufts Medical Center, Boston, MA 02111, USA

**Keywords:** inhaled treprostinil, pulmonary arterial hypertension, pulmonary hypertension associated with interstitial lung disease, tolerance predictors, right ventricular function, tricuspid annular plane systolic excursion, cardiac index, pulmonary vascular resistance, WHO functional class

## Abstract

**Background**: Treprostinil has demonstrated effectiveness in treating Pulmonary Arterial Hypertension (PAH) and Pulmonary Hypertension associated with Interstitial Lung Disease (PH-ILD). However, tolerability remains a clinical challenge. Identifying factors influencing tolerability is important, given the adverse outcomes of PAH and PH-ILD and the potential of treprostinil to slow disease progression. Objective: This study was undertaken to identify tolerance factors and develop a predictive scoring system. **Methods**: A retrospective analysis of 65 patients (37 PAH, 28 PH-ILD) was conducted using patient history, pulmonary function tests (PFTs), transthoracic echocardiograms (TTEs), and right heart catheterizations (RHCs). Of these, 67.7% (*n* = 44) tolerated treprostinil, while 32.3% (*n* = 21) were intolerant. **Results**: Patients who tolerated treprostinil had better pulmonary function, with a higher forced expiratory volume in one second/forced vital capacity (FEV_1_/FVC) ratio (82.27 ± 16.06 vs. 72.86 ± 17.76, *p* = 0.037) and superior right ventricular function, as indicated by higher tricuspid annular plane systolic excursion (TAPSE: 2.05 ± 0.37 vs. 1.64 ± 0.42, *p* < 0.001), higher cardiac index (CI: 2.51 ± 0.67 vs. 2.03 ± 0.53, *p* = 0.003), and improved functional status (*p* < 0.001). The Inhaled Treprostinil Intolerance Score (ITIS), incorporating TAPSE < 1.6, CI < 2, FEV_1_/FVC < 70%, and WHO functional class (FC) 3 or 4, demonstrated strong predictive accuracy (cutoff ≥ 2, AUC = 0.884 ± 0.048, *p* < 0.001). Predictive performance was stronger in PAH patients (AUC = 0.921 ± 0.053) than PH-ILD (AUC = 0.833 ± 0.093, *p* < 0.001). **Conclusions**: These findings demonstrate the importance of clinical parameters in predicting treprostinil tolerance. Further investigation is warranted to refine the scoring system, particularly for PH-ILD patients.

## 1. Background

Pulmonary hypertension (PH) encompasses a range of disorders characterized by elevated pulmonary artery pressures and progressive vascular remodeling, which leads to right ventricular dysfunction and eventual failure [1,2,3,4]. Among these conditions, pulmonary arterial hypertension (PAH) and pulmonary hypertension associated with interstitial lung disease (PH-ILD) are particularly challenging due to their overlapping symptoms with other diseases, requiring comprehensive evaluations for accurate diagnosis [5,6,7,8,9,10,11]. Both PAH and PH-ILD result in significant morbidity and mortality, primarily due to impaired hemodynamics, limited exercise capacity, and systemic complications [5,6,10,12,13,14,15].

The management of PAH—and more recently, PH-ILD—has improved with the use of prostacyclin analogues. Inhaled treprostinil has proven effective in enhancing quality of life and reducing mortality by decreasing pulmonary vascular resistance and improving cardiac output in both subgroups [16,17]. Administered via inhalation, treprostinil has been shown to be safe and effective, with fewer systemic side effects compared to intravenous or oral formulations [16]. However, the tolerability of inhaled treprostinil therapy may be influenced not only by its pharmacological effects but also by as yet undefined patient-specific factors.

Despite its efficacy, some patients have difficulty tolerating inhaled treprostinil due to adverse effects such as cough, throat irritation, and worsening clinical symptoms (e.g., increased shortness of breath, and worsening fatigue), common in both PAH and PH-ILD populations [18,19,20]. Given the poor prognosis associated with delayed treatment, early identification of patients at risk for poor tolerance is critical for preventing treatment discontinuation and improving patient outcomes [21,22]. Discontinuation of inhaled treprostinil has been linked to an increased risk of disease progression, particularly in patients with PAH, regardless of disease severity [22,23]. When patients cannot tolerate inhaled treprostinil, available treatment options become limited, creating a significant clinical challenge. Switching to alternatives, such as intravenous therapies, introduces logistical and safety concerns due to their invasive nature and higher risk of systemic complications [24,25,26,27,28]. This leaves clinicians with few viable options, particularly for patients with advanced disease, who already face a high symptom burden and limited functional capacity.

Thus, identifying patients at risk of intolerance to inhaled treprostinil before treatment initiation is potentially of great importance for optimizing therapeutic strategies and minimizing unnecessary risks. Predictive tools that incorporate patient-specific factors—such as clinical, functional, and hemodynamic variables—might help address this unmet need, enabling personalized treatment decisions and reducing the likelihood of treatment failure. This study was undertaken to identify factors contributing to intolerance to inhaled treprostinil, specifically by evaluating whether a composite score could predict such intolerance in patients with PAH and PH-ILD. To accomplish this, a simple, non-invasive tool was developed using data from patient history, pulmonary function tests (PFTs), transthoracic echocardiograms (TTE), and right heart catheterization (RHC).

## 2. Materials and Methods

We conducted a retrospective analysis of 65 patients (37 with PAH and 28 with PH-ILD) who underwent evaluation between 14 August 2016 and 30 September 2024 at Hartford Hospital (Hartford, CT, USA). Of these patients, 67.7% (*n* = 44) were classified as tolerant to inhaled treprostinil, while 32.3% (*n* = 21) were intolerant. Patients were identified using the International Classification of Diseases, 10th Revision (ICD-10) codes and all cases were subsequently reviewed and confirmed by a PH expert to ensure diagnostic accuracy. This study was approved by the Institutional Review Board (IRB) at Hartford HealthCare, ensuring adherence to ethical standards for patient data protection.

Patients were eligible if they were ≥18 years of age (no upper limit), of any gender, race, or ethnicity, had a confirmed diagnosis of PAH and/or PH-ILD, were evaluated and treated at Hartford Hospital between 14 August 2016 and 30 September 2024, and were prescribed inhaled treprostinil. Patients were excluded if they were <18 years of age, lacked a confirmed diagnosis of PAH or PH-ILD, or had not been prescribed inhaled treprostinil.

All study data were extracted from the electronic medical record (Epic). The following variables were collected: age, sex/gender, race/ethnicity, medical history (PAH or PH-ILD), World Health Organization functional class (WHO FC), PFTs parameters (forced expiratory volume in one second (FEV_1_), forced vital capacity (FVC), forced expiratory volume in one second/forced vital capacity (FEV_1_/FVC ratio), diffusing capacity of the lung for carbon monoxide (DLCO), and total lung capacity (TLC)), TTEs parameters (left ventricular ejection fraction [LVEF], estimated right atrial pressure [eRAP], right ventricular systolic pressure [RVSP], and tricuspid annular plane systolic excursion [TAPSE]), and RHC parameters (mean pulmonary artery pressure [mPAP], pulmonary capillary wedge pressure [PCWP], pulmonary vascular resistance [PVR], cardiac index [CI], and pulmonary artery pulsatility index [PAPi]).

Eligible patients met established diagnostic criteria for PAH or PH-ILD and had received inhaled treprostinil therapy. PAH was defined hemodynamically as a mean pulmonary arterial pressure (mPAP) ≥ 25 mmHg, pulmonary vascular resistance (PVR) ≥ 3 Wood units, and PCWP ≤ 15. PH-ILD was diagnosed based on the presence of interstitial lung disease confirmed by high-resolution computed tomography (HRCT) in conjunction with evidence of pulmonary hypertension on right heart catheterization, defined as mPAP ≥ 25 mmHg, PVR ≥ 3 Wood units, and PCWP ≤ 15 mmHg. These thresholds were applied in accordance with the prevailing diagnostic criteria and therapeutic indications for inhaled treprostinil during the majority of the study period. We recognize that the 2022 ESC/ERS guidelines have revised the hemodynamic definition of pre-capillary PH (mPAP > 20 mmHg, PVR > 2 WU, and PCWP ≤ 15 mmHg); however, inhaled treprostinil therapy continues to be indicated based on the prior thresholds, which were therefore used consistently for this analysis.

Treprostinil was administered either as an inhalation solution or as a dry powder inhaler. Dose titration for inhalation solution was performed in increments of 3 breaths, whereas dry powder inhalation was titrated in increments of 16 mcg. The target dose was typically 8–9 breaths four times daily, with clinical benefits generally emerging after approximately 16 weeks of treatment.

Tolerability was defined as the ability to maintain the prescribed treatment regimen and complete dose titration according to the recommended schedule (e.g., by 3 breaths for inhalation solution or 16 mcg for dry powder inhalation), with patients expected to reach the target of 8–9 breaths four times daily as described above. Patients were considered tolerant if they were able to achieve and maintain these dosing schedules without experiencing treatment-limiting adverse effects such as cough, throat irritation, worsening dyspnea, worsening fatigue, dizziness, or systemic prostacyclin-related symptoms (e.g., headache or flushing). Patients were classified as intolerant if any of the following occurred due to adverse effects or clinician-judged clinical worsening: (i) discontinuation of inhaled treprostinil; (ii) inability to complete at least one scheduled up-titration despite standard supportive measures; or (iii) inability to maintain dosing, including de-escalation or interruptions that prevented progression toward the target dose. Comparisons between the tolerant and intolerant groups were performed using categorical analyses. We conducted receiver operating characteristic (ROC) curve analysis to evaluate the predictive performance of the composite score. A categorical threshold of ≥2 was assessed. Subgroup analyses were performed to compare predictive accuracy between PAH and PH-ILD patients.

SPSS v. 29.0 (IBM; Armonk, NY USA, 2022) was used for all analyses.

## 3. Results

### 3.1. Patient’s Demographic and Clinical Characteristics

A total of 65 patients were included in this study, comprising 37 with PAH and 28 with PH-ILD. Of these, 44 patients (67.7%) were classified as tolerant to inhaled treprostinil, while 21 patients (32.3%) were classified as intolerant. When stratified by diagnosis, 23 (62.2%) of PAH patients were tolerant and 14 (37.8%) were intolerant, whereas 21 (75.0%) of PH-ILD patients were tolerant and 7 (25.0%) were intolerant. Baseline characteristics of the cohort are summarized in Table 1.

WHO FC was a significant determinant of tolerability. All patients in the intolerant group were classified as FC 3 or 4, whereas the tolerant group exhibited a more favorable distribution, with 13.6% in FC 1, 65.9% in FC 2, 6.8% in FC 3, and 13.6% in FC 4 (*p* < 0.001). These findings suggest an important role for functional status as a clinical marker of disease severity and treatment tolerability (Table 1).

In contrast, demographic variables, including age, gender, race, and ethnicity, were not significant predictors of tolerability. The mean age of patients in the intolerant group was 73.19 years (SD = 12.78) compared to 67.80 years (SD = 13.72). Among the intolerant group, 52.4% were male and 47.6% were female, whereas the tolerant group comprised 40.9% male and 59.1% female. The majority of patients in both groups were Caucasian (71.4% in the intolerant group vs. 81.8% in the tolerant group). Black/African American patients constituted 23.8% of the intolerant group and 9.1% of the tolerant group, while patients from other racial backgrounds represented 4.8% and 9.1%, respectively. Most patients were non-Hispanic (95.2% in the intolerant group vs. 90.9% in the tolerant group), with a small percentage identifying as Hispanic (4.8% vs. 9.1%) (Table 1).

### 3.2. Pulmonary Function Tests

The FEV_1_/FVC ratio was significantly lower in the intolerant group, with a mean of 72.86% (SD = 17.76%) compared to 82.27% (SD = 16.06%) in the tolerant group (*p* = 0.037; Table 2). A detailed evaluation revealed that 10 patients with an FEV_1_/FVC < 70 had a history of PAH, while 8 patients with an FEV_1_/FVC < 70 had a history of PH-ILD. Among the PAH patients with concomitant obstructive lung disease, it was postulated that the underlying etiology was predominantly related to an asthma-like phenotype rather than chronic obstructive pulmonary disease (COPD). This distinction was based on comprehensive clinical assessments, including symptomatology, response to bronchodilators, and the absence of significant smoking history in most cases. Conversely, all PH-ILD patients with obstructive lung disease exhibited features consistent with the combined pulmonary fibrosis and emphysema (CPFE) phenotype, a distinct and well-recognized subtype of ILD. CPFE is characterized by the coexistence of emphysematous changes and fibrotic remodeling, often leading to a unique clinical trajectory with increased susceptibility to pulmonary hypertension and adverse outcomes [29].

In contrast, other pulmonary function parameters did not achieve statistically significant differences between groups. Patients in the intolerant group had a mean FEV_1_ of 68.57% (SD = 20.55%), compared to 73.00% (SD = 20.51%) in the tolerant group. Similarly, the mean FVC was 74.33% (SD = 22.68%) in the intolerant group and 77.55% (SD = 23.72%) in the tolerant group. TLC was below that predicted in both groups, with the intolerant group averaging 76.10% (SD = 18.22%) and the tolerant group averaging 77.77% (SD = 22.62%). The DLCO was markedly reduced in both groups, with a mean DLCO of 41.38% predicted in the intolerant group (SD = 13.38%) and 47.55% predicted in the tolerant group (SD = 21.05%) (Table 2).

Multiple pulmonary function parameters, including FEV_1_, FVC, TLC, DLCO, and FEV_1_/FVC, were examined for their relationship with tolerability. In this cohort, FEV_1_ and FVC did not demonstrate statistically significant associations (*p* = 0.419 and *p* = 0.606, respectively), whereas FEV_1_/FVC did (*p* = 0.037). For this reason, FEV_1_/FVC was selected for inclusion in the ITIS score, while acknowledging that ERS/ATS 2022 guidelines recognize FEV_1_ and FEV_1_/VC as important measures of ventilatory impairment that may be better evaluated in larger or prospective cohorts.

### 3.3. Transthoracic Echocardiogram

TAPSE was significantly higher in the tolerant group, with a mean of 2.05 cm (SD = 0.37) compared to 1.64 cm (SD = 0.42) in the intolerant group (*p* < 0.001; Table 3).

Meanwhile, other echocardiographic parameters did not achieve statistically significant differences between groups. The eRAP was slightly higher in the tolerant group (5.95, SD = 4.49) compared to the intolerant group (4.24, SD = 2.77). The median eRAP was the same for both groups (3 mmHg), but the 75th percentile was higher in the tolerant group (8 mmHg) compared to the intolerant group (4 mmHg). LVEF values were slightly higher in the intolerant group (64.33%, SD = 7.28) compared to the tolerant group (62.02%, SD = 8.19). Similarly, the RVSP was marginally higher in the intolerant group (58.05 mmHg, SD = 21.71) compared to the tolerant group (56.43 mmHg, SD = 23.98) (Table 3).

### 3.4. Right Heart Catheterization

Table 4 summarizes RHC parameters between the tolerant and intolerant groups. CI was significantly higher in the tolerant group (mean = 2.51, SD = 0.67) compared to the intolerant group (mean = 2.03, SD = 0.53; *p* = 0.003; Table 4).

Apart from this, other RHC parameters did not achieve statistically significant differences between groups. Both groups exhibited elevated mean pulmonary artery pressure, with the tolerant group averaging 35.64 mmHg (SD = 12.91) and the intolerant group averaging 34.10 mmHg (SD = 8.75). The PCWP was similar between the groups, with slightly lower values in the tolerant group (10.16 mmHg, SD = 3.96) compared to the intolerant group (10.86 mmHg, SD = 4.00). The PAPi showed a modest trend toward lower values in the tolerant group (6.89, SD = 2.96) compared to the intolerant group (8.03, SD = 5.19). Lastly, PVR was slightly lower in the tolerant group (6.46 Wood units, SD = 4.02) compared to the intolerant group (7.40 Wood units, SD = 3.34) (Table 4).

### 3.5. Development of a Predictive Scoring System for Assessing Tolerance to Inhaled Treprostinil

We analyzed data from PFTs, TTE, and RHC to identify key clinical and hemodynamic parameters associated with tolerability. Among these parameters, TAPSE and CI were significantly lower in patients who were intolerant to inhaled treprostinil (TAPSE: *p* < 0.001; CI: *p* = 0.003).

**Figure 1:**
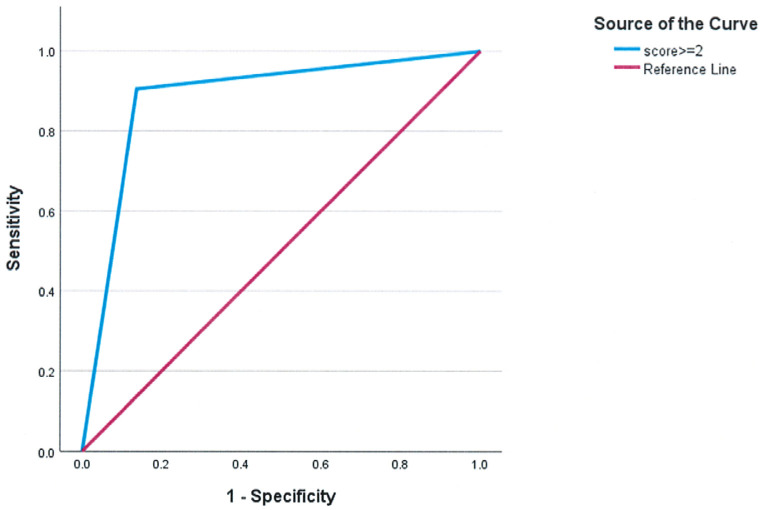
ROC curve of the composite score to identify intolerance to inhaled treprostinil. For clinical use, choosing a cutoff of ≥2 results in AUC = 0.884 ± 0.048, *p* < 0.001.

FC was also significantly associated with tolerability, as patients in FC 3 or 4 had a higher likelihood of intolerance (*p* < 0.001). Furthermore, patients who were intolerant to inhaled treprostinil had significantly worse pulmonary function, reflected in a FEV_1_/FVC ratio (72.86 ± 17.76 vs. 82.27 ± 16.06, *p* = 0.037). To facilitate clinical decision-making, these variables were incorporated into a composite assessment, the Inhaled Treprostinil Intolerance Score (ITIS), ranging from 0 to 4, with 1 point assigned for each of the following criteria: TAPSE < 1.6 cm, CI < 2.0 L/min/m^2^, WHO FC 3 or 4, and FEV_1_/FVC ratio < 70%. The predictive accuracy of the composite scoring system for identifying intolerance to inhaled treprostinil was evaluated using ROC curve analysis. ITIS demonstrated strong predictive accuracy, with an area under the curve (AUC) of 0.884 (SD = 0.048, *p* < 0.001) and a clinically significant threshold of ≥2 identifying intolerance. (Figure 1)

### 3.6. Predictive Performance in PAH and PH-ILD

**Figure 2:**
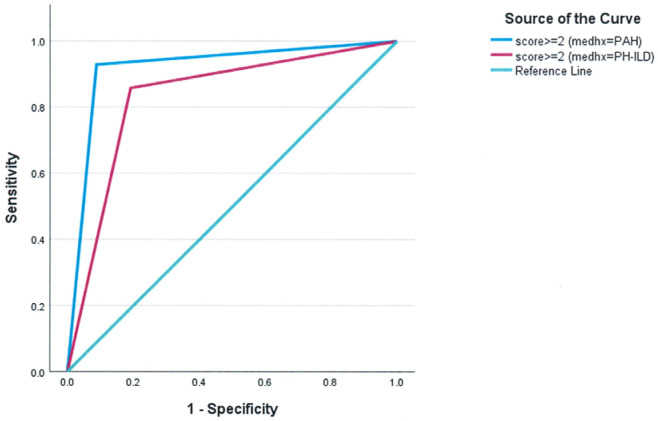
Subgroup analysis of the predictive performance of the composite score in PAH and PH-ILD. For clinical use, choosing a cutoff of ≥2 results in AUC = 0.921 ± 0.053, *p* < 0.001 for PAH (cs2GE2 (medhx = 0)) and AUC = 0.833 ± 0.093, *p* < 0.001 for PH-ILD (cs2GE2 (medhx = 1)).

The predictive performance of the score was assessed in both PAH and PH-ILD patient groups with internal validation conducted to ensure the robustness of the findings. A categorical threshold of ≥2 demonstrated strong predictive performance. In PAH, the AUC was 0.921 (SD = 0.053, *p* < 0.001), while in PH-ILD, it was 0.833 (SD = 0.093, *p* < 0.001) (Figure 2).

## 4. Discussion

This analysis identified key clinical and hemodynamic parameters that predict the tolerability of inhaled treprostinil in patients with PAH or PH-ILD. Patients were categorized as either tolerant (*n* = 44, 67.7%) or intolerant (*n* = 21, 32.3%), with significant differences observed in baseline characteristics and hemodynamics between the two groups, as summarized in Table 5. These distinctions contributed to the development of a scoring system that accurately predicted intolerance to treatment.

Inhaled treprostinil plays an important role in the management of both PAH and PH-ILD, significantly improving quality of life for patients who can tolerate the therapy. However, discontinuation due to adverse effects remains a significant challenge. Previous studies report a discontinuation rate of 24–41% among PAH patients [19,20], mostly due to side effects. Side effects pose a particular challenge for patients with PH-ILD; in the INCREASE trial, 40 out of 163 patients (approximately 25%) discontinued treatment prematurely, with 16 (40%) citing adverse events as the primary reason for discontinuation [18]. The post hoc analysis from that study underscored the importance of adherence for therapeutic success, as benefits typically emerge after approximately 16 weeks of treatment, with successful outcomes closely tied to achieving 8–9 doses [30].

Our findings are consistent with these observations, with 32.3% of patients in our cohort experiencing treatment-limiting intolerance. Factors such as lower FEV_1_/FVC ratios on PFTs, higher FC, and markers of right ventricular (RV) dysfunction (e.g., reduced TAPSE and CI) were associated with decreased tolerability to inhaled treprostinil. These findings suggest that patients with evidence of obstructive lung disease or advanced pulmonary hypertension, especially those with impaired RV function, are less likely to tolerate inhaled treprostinil. This aligns with results from the PERFECT trial, which was prematurely terminated due to poor outcomes in PH-COPD patients treated with inhaled treprostinil [31]. Importantly, the post hoc analysis of the study identified specific hemodynamic and pulmonary function thresholds that could help refine patient selection for future trials. Patients with mPAP > 40 mmHg and FEV_1_ > 40% appeared more likely to demonstrate benefit, whereas those with DLCO < 25% had a higher likelihood of poor outcomes. These findings reinforce the necessity of carefully selecting PH patients who are more likely to tolerate and respond to inhaled treprostinil therapy, ensuring that treatment is targeted to those most likely to benefit while minimizing the risk of early discontinuation and adverse events [32].

Taken together, these findings emphasize the necessity for a personalized approach to PH therapy. Individualized assessments are crucial for predicting the risk of early discontinuation and guiding treatment decisions. To aid in this, we developed the ITIS, which integrates four key clinical variables: TAPSE, CI, FEV_1_/FVC ratio, and FC. In our cohort, ITIS demonstrated an excellent AUC of 0.884 with a threshold of ≥2 in the overall cohort, highlighting its strong predictive accuracy. ITIS provides a straightforward and practical approach to identifying patients at risk for intolerance to inhaled treprostinil, allowing for more personalized treatment strategies and potentially reducing the risk of treatment failure. Moreover, this scoring system offers a robust method for identifying high-risk patients who may benefit from additional interventions or closer monitoring, ensuring a more tailored approach to therapy.

The ITIS score assigns equal weight to each variable for simplicity and clinical usability. While differential weighting could be derived using more complex modeling techniques, our small sample size limited such approaches. Future studies with larger datasets may allow for refinement of the score using regression-based or machine-learning-derived weights, similar to methods employed in the development of scores like REVEAL.

Notably, the score performed better in PAH than in PH-ILD across both approaches. This difference may reflect variations in disease pathophysiology, clinical presentation complexity in PH-ILD, or differences in the underlying data used for prediction. While PAH and PH-ILD represent distinct clinical entities, they share overlapping treatment strategies and therapeutic challenges in real-world practice. The decision to analyze them together was driven by our sample size limitations and the clinical relevance of inhaled treprostinil in both subgroups. Nonetheless, we recognize this as a potential limitation and conducted subgroup analyses to explore predictive performance separately in each population. Despite these differences, the ITIS remains a tool for risk stratification in both patient populations, aiding in the early identification of individuals at higher risk of intolerance to inhaled treprostinil.

Our study underscores the importance of patient-specific factors in guiding the use of inhaled treprostinil for PAH and PH-ILD. The ITIS offers means to personalize treatment strategies, allowing for early identification of patients at high risk of intolerance and facilitating more effective management. However, further validation in larger, prospective cohorts is warranted to confirm these findings and optimize the score for broader clinical application.

## 5. Limitations

Several limitations should be considered when interpreting the results of this study. First, the sample size was relatively small, necessitating the use of a pooled cohort to achieve adequate statistical power. This may limit the generalizability of the findings to larger, more diverse populations. Additionally, unmeasured confounding factors that could influence tolerability were not accounted for in our analysis, potentially affecting the validity of our results. The inclusion criteria for this study, which required patients to adhere to the prescribed treatment regimen and tolerate dose titration, may have excluded individuals who could have succeeded with multiple attempts or benefited from a lower dose. Furthermore, the method of inhaled treprostinil delivery, whether as a dry powder inhaler such as Yutrepia or as an inhalation solution like Tyvaso, was not explicitly examined and may have influenced tolerability and discontinuation rates. Although the study reported that 75.0% of PH-ILD patients tolerated therapy compared to 62.2% of PAH patients, this apparent difference in tolerance was not formally tested for statistical significance and should be interpreted cautiously. Additionally, patients were initially identified using ICD-10 codes; however, all diagnoses were subsequently reviewed and confirmed by PH experts using accompanying clinical and hemodynamic data, which reduces the risk of misclassification bias.

Another consideration is that although mean PCWP values were below 15 mmHg, several patients had values in the upper normal range (up to 14 mmHg). While patients with overt left-sided heart failure were excluded, we cannot exclude the possibility that some had underlying cardiac comorbidities (e.g., diastolic dysfunction, atrial fibrillation, or valvular disease) contributing to borderline elevations in filling pressures, raising the potential for mixed pulmonary hypertension. Future prospective studies with detailed cardiac phenotyping would be necessary to clarify this issue.

Although FEV_1_/FVC was the only pulmonary function parameter achieving statistical significance in our sample, ERS/ATS 2022 technical standards emphasize a comprehensive physiologic approach, recognizing that parameters such as FEV_1_ or FEV_1_/VC may be more sensitive markers of ventilatory impairment. Future studies with larger or prospective datasets should evaluate these alternative parameters as potential refinements to the ITIS model.

Due to limited sample size, subgroup-specific scoring systems for PAH and PH-ILD were not feasible, though future studies may benefit from stratified model development. Although multivariable regression or machine-learning approaches may enhance model sophistication, our sample size precluded their use in this initial development study. Finally, the score was developed and then internally validated within the same cohort of patients, and external validation in a multi-center approach is necessary for continued evolution of the score. As the score was developed and internally tested within the same dataset, its apparent predictive strength may be inflated. External validation in independent cohorts is necessary to confirm generalizability and mitigate the risk of overfitting.

## 6. Conclusions

This study assessed the predictive performance of clinical metrics and composite scores in patients with PAH and PH-ILD, focusing on tolerance to inhaled treprostinil. Key factors such as TAPSE, CI, and FC were significantly associated with treatment tolerance. Specifically, lower TAPSE and CI values correlated with reduced tolerance (*p* < 0.001 and *p* = 0.003, respectively), and higher FC (class 3 or 4) strongly predicted intolerance (*p* < 0.001). Additionally, the FEV_1_/FVC ratio was significant (*p* = 0.037), further emphasizing the importance of both pulmonary and right ventricular function in treatment decisions.

The findings from this study provide a practical tool for clinicians to anticipate intolerance to inhaled treprostinil, enabling early intervention and more personalized management strategies. Moreover, these insights have broader implications for improving patient outcomes in PH care by aligning treatments with individual patient profiles.

The composite score developed in this study demonstrated strong predictive accuracy for intolerance, with AUC values exceeding 0.8 overall. Notably, in subgroup analyses, the AUC exceeded 0.9 in PAH and was greater than 0.8 in PH-ILD, further underscoring the robustness of the model across different patient populations. These findings reveal a critical gap in our understanding of the factors influencing the tolerability of inhaled treprostinil and highlight the need for further investigation, particularly in larger, more diverse cohorts. Additionally, refining the methodology to incorporate longitudinal data could enhance the predictive capacity and clinical utility of the scoring system.

A deeper understanding of the relationship among lung function, right ventricular performance, and hemodynamic parameters will be essential for optimizing treatment strategies and improving patient outcomes. Ongoing research in this area will help refine therapeutic approaches for these complex and challenging conditions.

## Figures and Tables

**Table 1:** Baseline characteristics of inhaled treprostinil tolerant and intolerant patients.

Variable	All Patients (*n* = 65)	Tolerant Group (*n* = 44)	Intolerant Group (*n* = 21)	*p* Value
**Patient Demographics**				
Age (Mean ± SD, years)	69.54 ± 13.57	67.80 ± 13.72	73.19 ± 12.78	0.135
Male gender % (*n*)	44.6% (29)	40.9% (18)	52.4% (11)	0.432
Race % (*n*) White Black/African American Other	78.5% (51) 13.8% (9) 7.7% (5)	81.8% (36) 9.1% (4) 9.1% (4)	71.4% (15) 23.8% (5) 4.8% (1)	0.251
Ethnicity % (*n*) Non-Hispanic Hispanic	92.3% (60) 7.7% (5)	90.9% (40) 9.1% (4)	95.2% (20) 4.8% (1)	1.000
WHOFC% (*n*) Class I Class II Class III Class IV	9.2% (6) 44.6% (29) 21.5% (14) 24.6% (16)	13.6% (6) 65.9% (29) 6.8% (3) 13.6% (6)	0.0% (0) 0.0% (0) 52.4% (11) 47% (10)	<0.001

Value in red denotes statistical significance at *p* < 0.05.

**Table 2:** Comparison of pulmonary function test parameters in patients tolerant and intolerant to inhaled treprostinil.

Variable	Tolerant Group (*n* = 44)	Intolerant Group (*n* = 21)	*p* Value
FEV_1_ (Mean ± SD, L)	73.00 ± 20.51	68.57 ± 20.55	0.419
FVC (Mean ± SD, L)	77.55 ± 23.72	74.33 ± 22.68	0.606
FEV_1_/FVC (% ± SD, %)	82.27 ± 16.06	72.86 ± 17.76	0.037
DLCO (Mean ± SD, mL/min/mmHg)	47.55 ± 21.05	41.38 ± 13.38	0.225
TLC (Mean ± SD, L)	77.77 ± 22.62	76.10 ± 18.22	0.768

Value in red denotes statistical significance at *p* < 0.05.

**Table 3:** Comparison of transthoracic echocardiogram parameters in patients tolerant and intolerant to inhaled treprostinil.

Variable	Tolerant Group (*n* = 44)	Intolerant Group (*n* = 21)	*p* Value
eRAP (Mean ± SD, mmHg)	5.95 ± 4.49	4.24 ± 2.77	0.346
TAPSE (Mean ± SD, cm)	2.05 ± 0.37	1.64 ± 0.42	<0.001
LVEF (Mean ± SD, %)	62.02 ± 8.19	64.33 ± 7.28	0.275
RVSP (Mean ± SD, mmHg)	56.43 ± 23.98	58.05 ± 21.71	0.794

Value in red denotes statistical significance at *p* < 0.05.

**Table 4:** Comparison of right heart catheterization parameters between patients tolerant and intolerant to inhaled treprostinil.

Variable	Tolerant Group (*n* = 44)	Intolerant Group (*n* = 21)	*p* Value
mPAP (Mean ± SD, mmHg)	35.64 ± 12.91	34.10 ± 8.75	0.623
PCWP (Mean ± SD, mmHg)	10.16 ± 3.96	10.86 ± 4.00	0.510
PAPi (Mean ± SD, mmHg)	6.89 ± 2.96	9.03 ± 5.19	0.263
CI (Mean ± SD, L/min/m^2^)	2.51 ± 0.67	2.03 ± 0.53	0.003
PVR (Mean ± SD, Wood units)	6.46 ± 4.02	7.40 ± 3.34	0.358

Value in red denotes statistical significance at *p* < 0.05.

**Table 5:** Summary of statistical findings.

Variable	Tolerant Group (*n* = 44)	Intolerant Group (*n* = 21)	*p* Value
TAPSE (Mean ± SD, cm)	2.05 ± 0.37	1.64 ± 0.42	<0.001
CI (Mean ± SD, L/min/m^2^)	2.51 ± 0.67	2.03 ± 0.53	0.003
FEV_1_/FVC (% ± SD, %)	82.27 ± 16.06	72.86 ± 17.76	0.037
WHOFC % (*n*) Class I Class II Class III Class IV	13.6% (6) 65.9% (29) 6.8% (3) 13.6% (6)	0.0% (0) 0.0% (0) 52.4% (11) 47.0% (10)	<0.001

Values in red denotes statistical significance at *p* < 0.05.

## Data Availability

The data presented in this study are available on request from the corresponding author due to ethical restrictions and institutional review board policies protecting patient confidentiality.
